# Genus *Echium* L.: Phytochemical Characterization and Bioactivity Evaluation for Drug Discovery

**DOI:** 10.3390/plants14162548

**Published:** 2025-08-15

**Authors:** Parvaneh Sheydaei, Maria Emília Amaral, Ana Paula Duarte

**Affiliations:** 1 RISE, Health Sciences Research Centre (CICS), University of Beira Interior, 6200-506 Covilhã, Portugal; apcd@ubi.pt; 2 Fiber Materials and Environmental Technologies Research Unit (FibEnTech-UBI), University of Beira Interior, Rua Marquês D’Ávila e Bolama, 6201-001 Covilhã, Portugal; mecca@ubi.pt

**Keywords:** *Echium* spp., ethnomedicinal uses, pharmacological studies, phytochemistry

## Abstract

*Echium* L. is a genus of flowering plants from the Boraginaceae family that includes several species traditionally used in herbal medicine. *Echium* spp. have been applied for treating wounds, urinary tract infections, inflammation, respiratory ailments, cardiovascular disorders, and microbial infections. The roots and flowers are most frequently used, typically prepared as decoctions or infusions. Phytochemical studies have identified diverse bioactive compounds, including phenolics, naphthoquinones, shikonins, fatty acids, sterols, terpenoids, amino acids, and toxic pyrrolizidine alkaloids. Reported pharmacological effects include antioxidant, antimicrobial, and cytotoxic activities, primarily attributed to polyphenolic and terpenoid content. However, the presence of toxic alkaloids also raises concerns regarding safety. This review provides a comprehensive overview of the ethnomedicinal uses, phytochemical components, and pharmacological activities of *Echium* species. The bioactivities observed in genus *Echium* L. substantiate the necessity for preclinical and clinical investigations to thoroughly elucidate and validate the therapeutic potential of this genus and emphasize its relevance in the development of novel therapeutic agents.

## 1. Introduction

In recent years, there has been a substantial increase in the use of natural products for managing various diseases, primarily driven by their favorable safety profiles, economic accessibility, and widespread availability [1,2]. Although numerous studies have explored the pharmacological potential of bioactive compounds derived from *Echium* species, a thorough evaluation of their therapeutic efficacy, safety concerns, and ethnomedicinal applications remains insufficiently addressed [3,4,5,6].

The genus *Echium* L. comprises approximately 67 recognized species predominantly distributed across Europe, North Africa, and the Macaronesian archipelago, with several species extending into Western Asia. Due to their remarkable ecological adaptability, *Echium* plants have been introduced to diverse regions worldwide [7,8]. For instance, Tunisia hosts eleven native species [9], Turkey contains nine distinct species, including *E. italicum*, *E. vulgare*, and *E. plantagineum* [10], while Egypt reports seven native taxa [11]. In Iran, *E. amoenum* (Persian borage), a biennial or perennial species from the Boraginaceae family, is widely utilized in traditional medicine. Typically found at altitudes ranging from 60 to 2200 m, it is consumed as an herbal infusion for managing the common cold, sore throat, gastrointestinal discomfort [6], and neurological disorders [3]. Its documented effects include sedative, analgesic [5], diuretic [4], antioxidant, and anxiolytic activities [12,13]. Beyond Iran, other species such as *E. italicum* are traditionally used in Turkey as teas and decoctions with diuretic and sedative effects [14]. Topical applications of powdered leaves or root extracts [15], either raw or formulated with beeswax and oils [16,17,18]. Additionally, *E. plantagineum* L. is used as a diuretic and diaphoretic through decoction [19], whereas *E. vulgare* L. and *E. russicum* J.F. are employed in the treatment of wound healing disorders through ointments [20]. In Serbia, *E. vulgare* is known for its antitussive, aphrodisiac, demulcent, diaphoretic, diuretic, pectoral, and vulnerary properties and its use in snakebite treatment [21]. Collectively, *Echium* species exhibit an impressive spectrum of pharmacological activities including antioxidant [12,21,22,23,24], anti-inflammatory [25], antiproliferative [26,27,28], antidepressant [29,30,31], anxiolytic [13,32,33,34], antiviral [35,36], antibacterial [37], analgesic [5], anticonvulsant [38], and lipid-regulating properties [39,40].

These effects are largely attributed to key phenolic compounds such as shikonin and other naphthoquinones, as well as essential fatty acids like linolenic acid, which are discussed in detail in subsequent sections. However, the presence of toxic constituents, particularly pyrrolizidine alkaloids (PAs), raises safety concerns. Notably, *E. plantagineum*, known as Salvation Jane or Paterson’s Curse, has been flagged by Australian and New Zealand food safety authorities due to its high PA content. Compounds such as echimidine, echiumine, acetylechimidine, 7-O-acetyl lycopsamine, and 7-O-acetyl intermedine have led to dietary recommendations limiting the consumption of honey derived from this plant to no more than two tablespoons per day [41]. Furthermore, the concentration and composition of these toxic alkaloids vary significantly depending on the plant’s geographical origin [42]. For example, while PAs such as senecionine, echimidine, and lycopsamine have been detected in *Echium-based* honey from Spain [43], no such compounds have been reported in Portuguese samples [41]. These findings underscore the pivotal role of environmental factors such as climate, soil composition, humidity, and pest prevalence in shaping the phytochemical profiles of *Echium* species, ultimately influencing their therapeutic efficacy and toxicity [44]. Supported by both traditional knowledge and emerging scientific evidence, the *Echium* genus represents a pharmacologically valuable group of plants with significant biomedical potential. This literature review seeks to provide an integrative synthesis of the medicinal applications, phytochemical diversity, pharmacological properties, and toxicological risks associated with various *Echium* species. Additionally, by compiling ethnopharmacological data, this work aims to inform future research directions toward the evidence-based use of *Echium* spp. in modern phytotherapy. The paper concludes with a critical appraisal of current findings and suggestions for future scientific exploration.

### Aims and Methodology

This review provides an overview of the plant’s ethnomedical applications, phytochemistry, pharmacological properties, and toxicological risks associated with various *Echium* species.

Methodology: The literature analysis necessitated that reports be disseminated in the English language, without imposing any exclusion criteria based on the publication date. Online databases like PubMed, Google Scholar, Web of Science, and Scopus were used to gather information using keywords such as *Echium* genus, ethnomedicinal uses, phytochemistry, bioactivities, and pharmacological activities. Chemical structures were drawn using the online Chemical JS, version 25.0.1 (PerkinElmer, Waltham, MA, USA 2025) ChemDraw.

## 2. Phytochemical Characterization

### 2.1. Polyphenolic Compound Extraction and Phenolic Profiles in Echium Species: Key Findings and Analytical Insights

**Figure 1:**
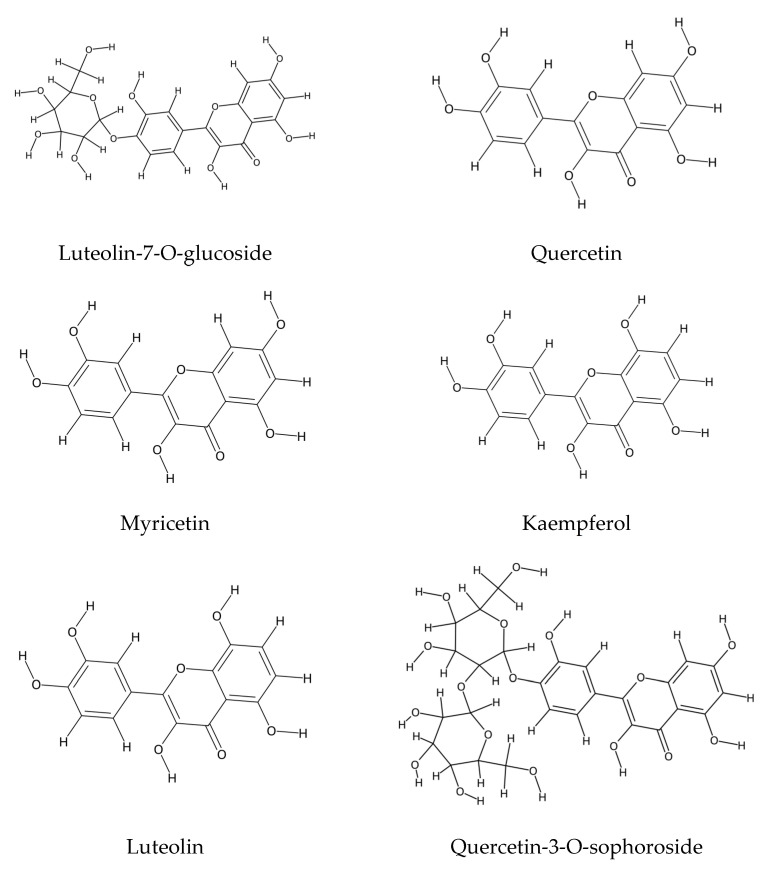

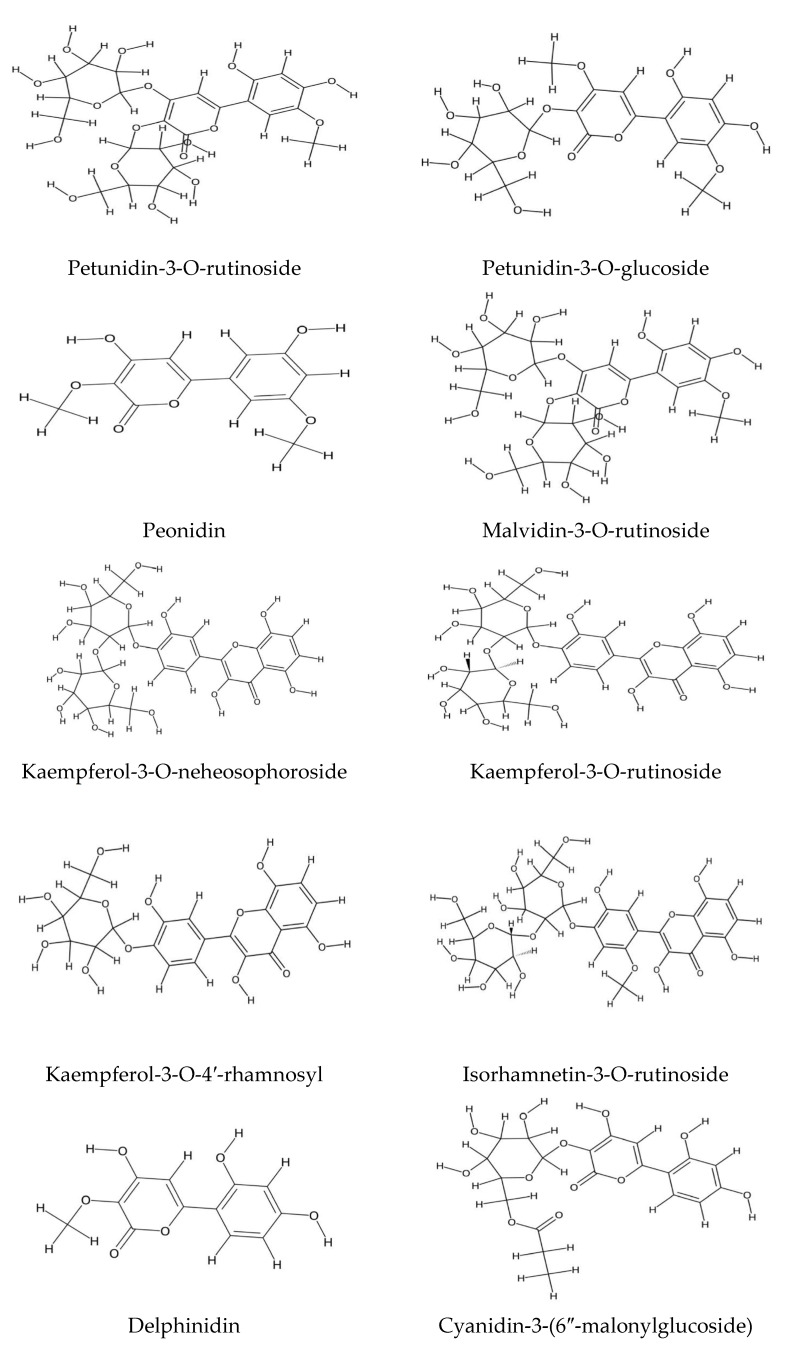

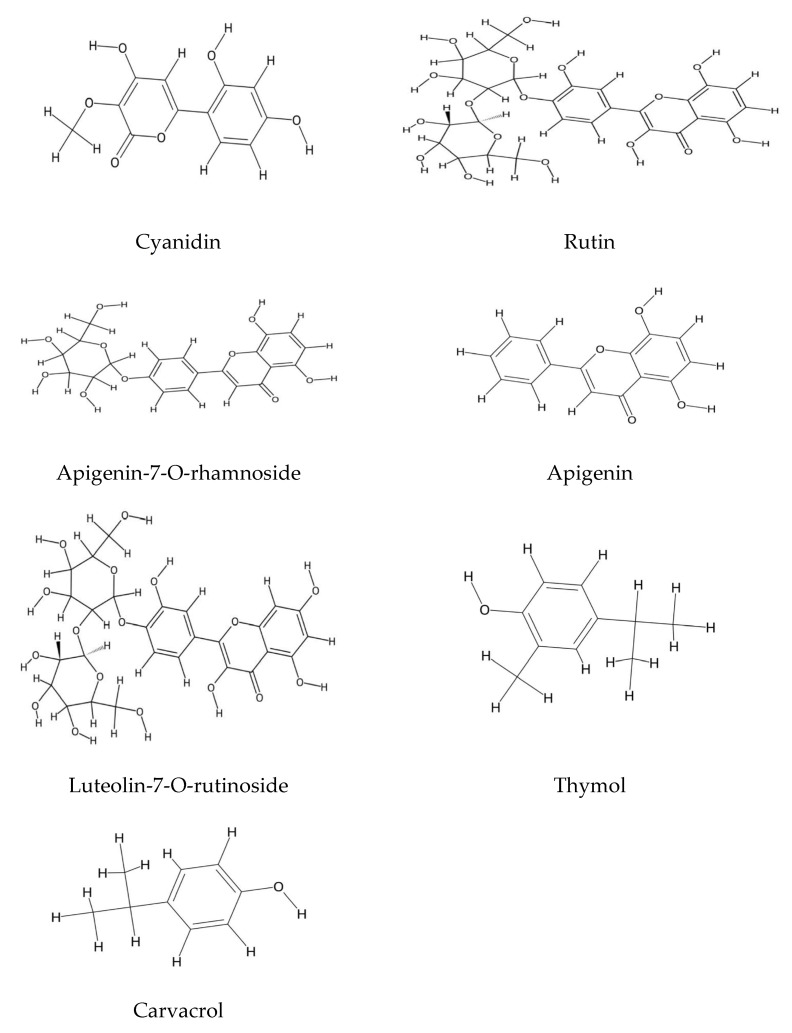
Phenolic compounds in *Echium* spp.

The genus *Echium* L. is renowned for its chemically diverse flavonoid profile, which has been rigorously investigated through the application of advanced analytical methodologies. These studies have unveiled a wide array of bioactive flavonoid compounds distributed across various species within the genus, as summarized below:

In *E. arenarium*, reverse-phase high-performance liquid chromatography (RP-HPLC) has identified several major flavonoids, including luteolin-7-O-glucoside, myricitrin, myricetin, and quercetin. Among these, luteolin-7-O-glucoside emerged as the most abundant and biologically active compound, underscoring its phytochemical relevance and potential therapeutic significance [45] (see Figure 1).

In other *Echium* species, an extensive range of flavonoid compounds has also been detected through HPLC–DAD–MS. These include kaempferol, peonidin, cyanidin, and malvidin, typically occurring as mono- or disaccharide derivatives glycosylated at the C3 position. Particularly noteworthy among these is kaempferol-3-O-neohesperidoside, a compound recognized for its notable bioactivity and pharmacological potential [46,47,48].

A comprehensive phytochemical investigation of *E. sericeum*, employing proton nuclear magnetic resonance (^1^H-NMR) and fast atom bombardment mass spectrometry (FAB-MS), led to the identification of four predominant flavonoids: apigenin, luteolin-7-O-rutinoside, apigenin-7-O-rhamnoside, and quercetin-3-O-rhamnoside (refer to Figure 1).

These findings collectively highlight the critical role of sophisticated spectroscopic techniques in the structural characterization and profiling of flavonoids in plant matrices [11].

**Figure 2:**
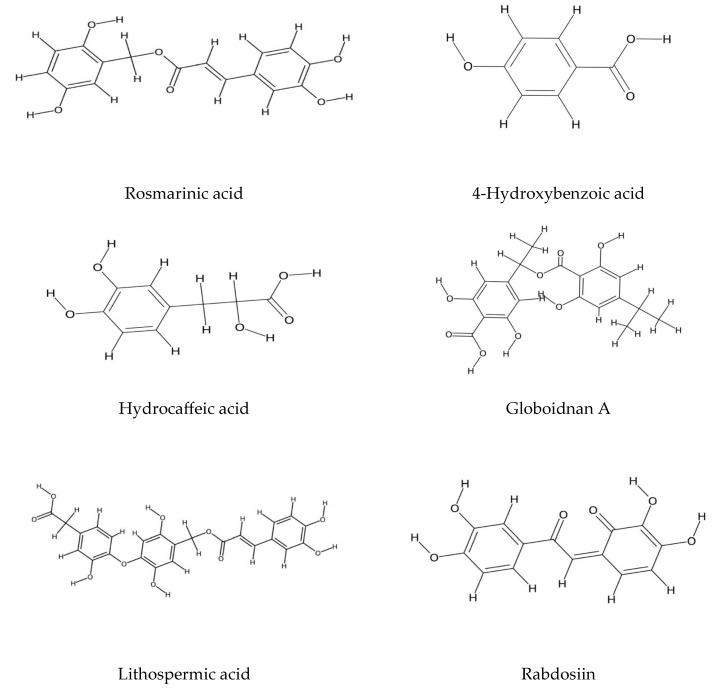

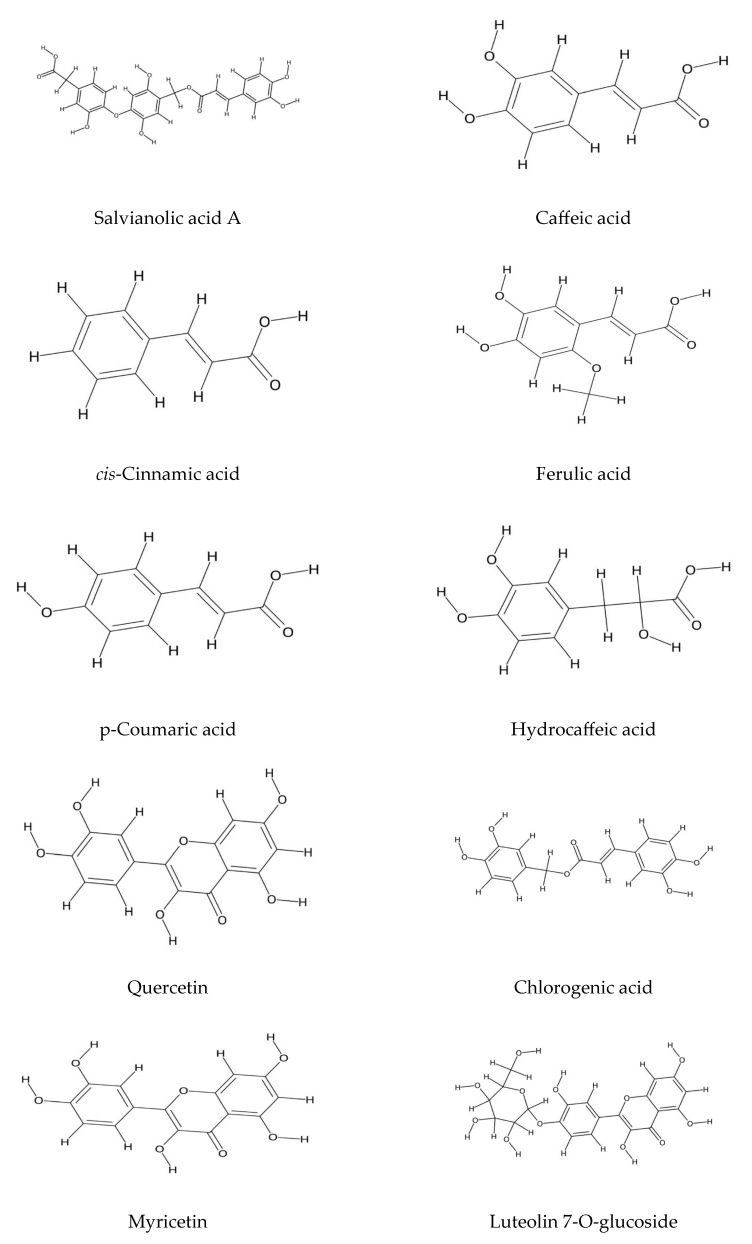

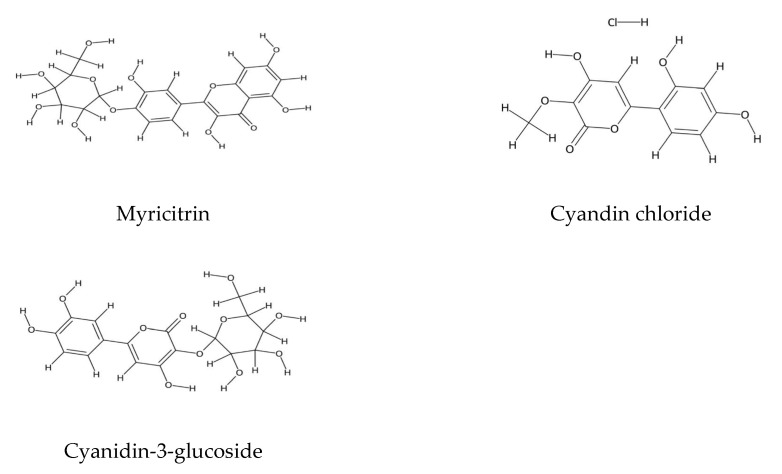
Phenolic acids in *Echium* spp.

Research on polyphenols shows that the extraction efficiency of these bioactive compounds is greatly affected by key factors, including extraction time, temperature, solvent-to-solid ratio, and the water content of the solvent [49,50,51,52,53]. Among the anthocyanins evaluated, cyanidin-3-glucoside yielded the highest concentration, followed in descending order by cyanin chloride, cyanidin-3-rutinoside, and pelargonidin-3-glucoside chloride. These results were obtained using a natural deep eutectic solvent composed of choline chloride and glycerol (CHGLY), which showed superior extraction performance compared to conventional solvents such as methanol, ethanol, and water [53] (see Figure 2).

Reverse-phase high-performance liquid chromatography (RP-HPLC) analysis of the ethyl acetate extract derived from *Echium arenarium* flowers revealed the presence of four major phenolic constituents: luteolin-7-O-glucoside (60.56 μg/mg of dry extract (DE)), myricitrin (27.92 μg/mg DE), myricetin (7.34 μg/mg DE), and quercetin (1.27 μg/mg DE) [45].

The essential oil derived from the hydrodistillation of the flowering aerial parts of *E. amoenum* (Fisch. & C. A. Mey)., identified thymol and carvacrol as the predominant monoterpenes [54]. Furthermore, rosmarinic acid has been consistently reported as the primary phenolic compound in *E. amoenum*, *E. russicum*, and *E. vulgare* [55,56,57,58]. Notably, hot water extractions of *Echium* species yield significantly higher concentrations of rosmarinic acid compared to other extraction methods [59].

Additional phenolic compounds identified in *E. russicum* include salvianolic acid A, rabdosiin, lithospermic acid, and eritrichin (also known as globoidnan A), enriching the phytochemical profile of the species [56] (refer to Figure 2).

A novel compound (caffeoyl derivative) has recently been identified in the leaves of *E. amoenum* using NMR spectroscopy [60]. This compound is considered an analog of rabdosin, a phytochemical previously reported in *E. russicum* [56] and *E. plantagineum* [61].

### 2.2. Echium Roots: Focus on Alkanin and Shikonin Compounds

**Figure 3:**
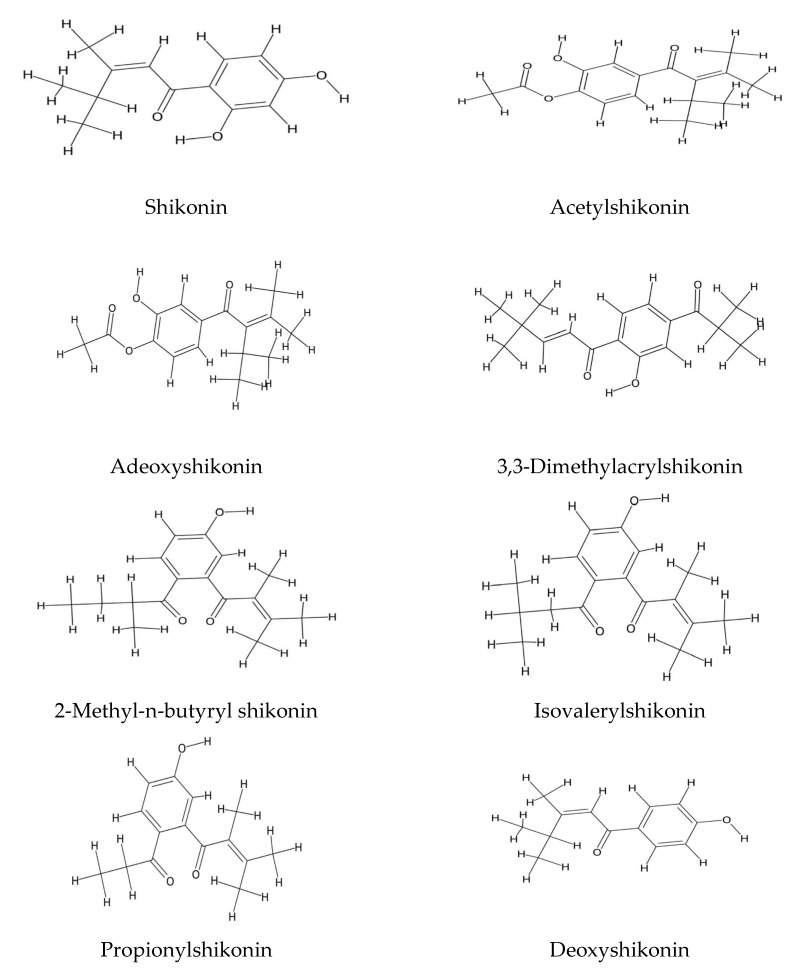

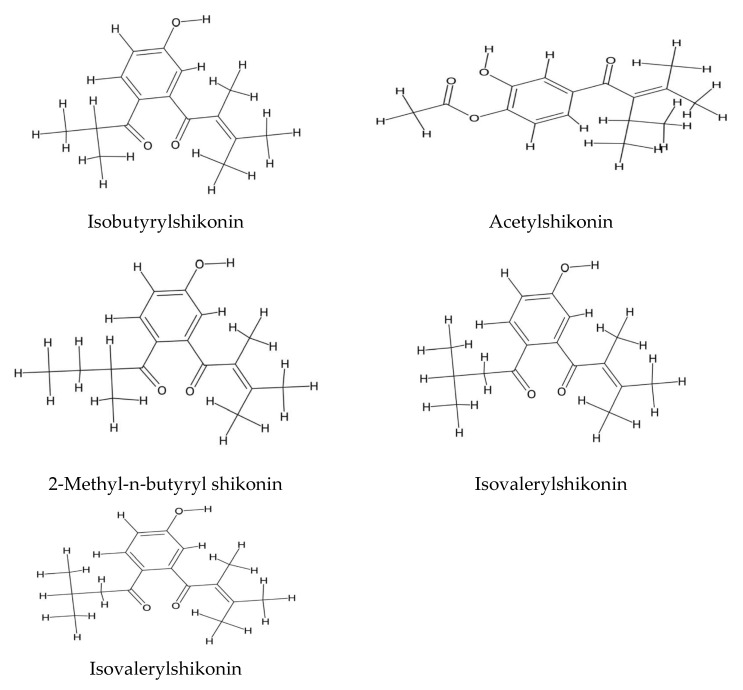
Naphthoquinones of *Echium* spp.

The roots of various *Echium* species are abundant in pharmacologically active naphthoquinones, notably alkanins and shikonins. These compounds are extensively documented for their wound-healing efficacy [10,62,63], alongside their anti-inflammatory and antimicrobial activities [64]. Among them, the ethanolic root extract of *E. italicum*, rich in naphthoquinone derivatives such as acetylshikonin, deoxyshikonin, and isovalerylshikonin, was shown to significantly elevate hydroxyproline levels in experimental mice, indicating enhanced collagen synthesis and tissue regeneration [10] (Figure 3). Similarly, the ether extracts of *Lithospermum erythrorhizon* and *Macrotomia euchroma* roots (both from the Boraginaceae family) have demonstrated wound-healing activity by stimulating granulation tissue formation in murine models [65]. In *Arnebia densiflora* and *Arnebia nobilis*, compounds like arnebin-1 have been implicated in wound contraction and accelerated re-epithelialization, further supporting the pivotal role of root-derived naphthoquinones in tissue repair [66,67]. Environmental factors such as seasonality and altitude markedly influence the biosynthesis of these compounds. For instance, roots of *E. plantagineum* harvested during the summer contain significantly higher levels of shikonins compared to those collected in winter or spring [68,69]. Additionally, roots from low-altitude areas yield three to five times more shikonins than those from higher elevations [70]. Beyond wound healing, shikonins, alkanins, and related naphthazarins exhibit a broad range of biological activities. These include strong antioxidant, antihelminthic, and purgative properties, as well as the induction of apoptosis in prostate cancer [63,71,72,73,74] and acute myeloid leukemia cells [75]. They also display potent antibacterial effects, especially against Gram-negative bacteria [59], and have shown neuroprotective effects in Alzheimer’s disease models [76]. Moreover, these compounds act as powerful allelopathic agents, inhibiting the growth and viability of competing plants, fungi, insects, and bacteria [77,78,79].

The remarkable therapeutic potential of naphthoquinone compounds, particularly shikonins and their derivatives, underscores the importance of *Echium* and related Boraginaceae species in biomedical research. Understanding the environmental and seasonal factors that influence their biosynthesis can guide the optimization of phytopharmaceutical production and support the development of novel, nature-based therapeutic strategies in wound management and oncology [21,80,81].

### 2.3. Steroids and Polyunsaturated Fatty Acids

**Figure 4:**
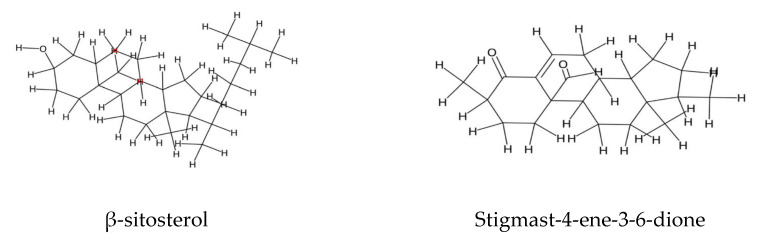
Sterons of *Echium* spp.

Two steroids, Stigmast-4-ene-3,6-dione and β-sitosterol, have been identified in the flowers of *E. vulgare*, with β-sitosterol exhibiting notable antioxidant properties [82,83] (Figure 4).

In the essential oil obtained via hydrodistillation from the aerial parts of *E. italicum*, 22 chemical constituents have been identified, with 1-hexadecanol and pulegone, a monoterpene ketone, emerging as the most abundant compounds [84]. Similarly, in the essential oil of *E. amoenum*, n-hexadecane (8.7%) and n-pentadecane (5.6%) are the predominant alkanes [85].

**Figure 5:**
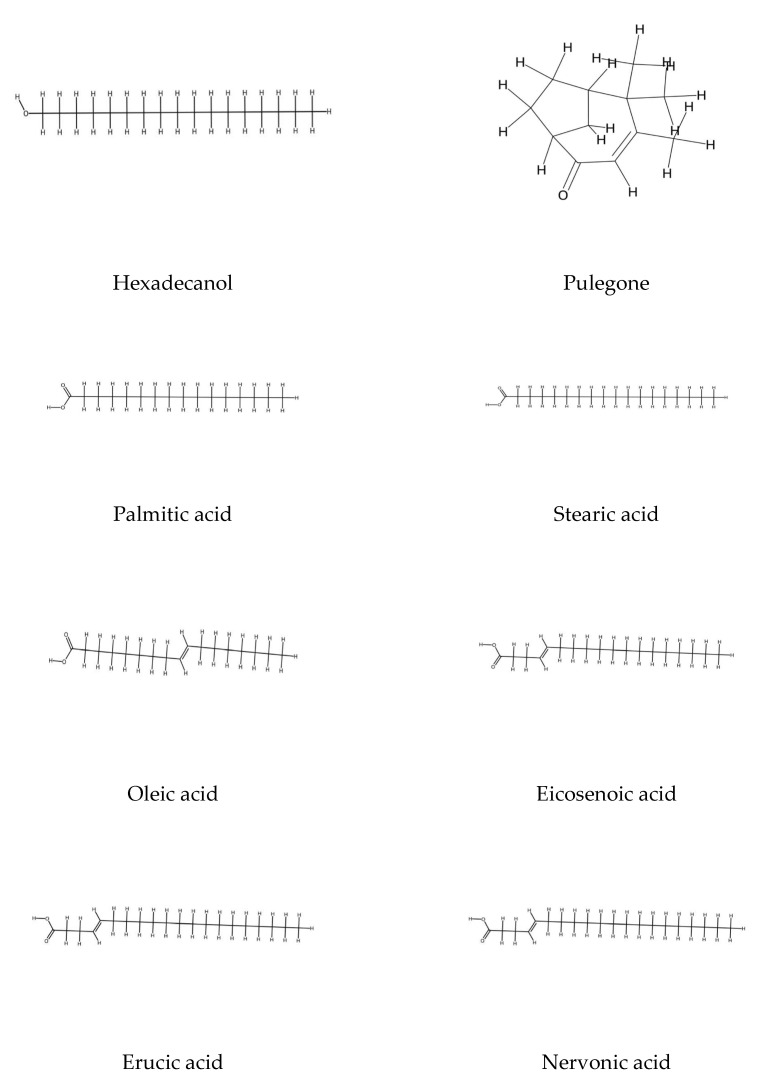

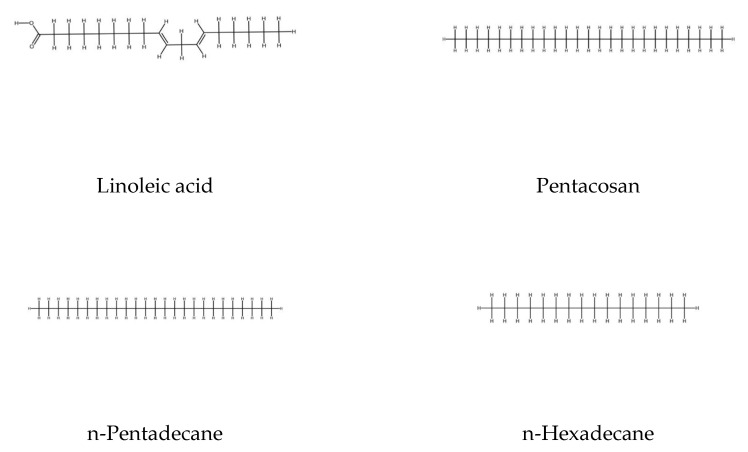
Fatty acids and hydrocarbon compounds present in essential and seed oils of *Echium* spp.

The seed oil of *E. amoenum* is particularly rich in polyunsaturated fatty acids (PUFAs), most notably α-linolenic acid (ALA), which constitutes approximately 40–41% of total fatty acids, followed by linoleic acid (19–20%). These PUFAs significantly outnumber the monounsaturated fatty acids (MUFAs), positioning *Echium* species as valuable botanical sources of essential fatty acids. Notably, among various *Echium* species, the highest concentration of ALA has been consistently reported in *E. amoenum* seed extracts [86] (Figure 5).

**Figure 6:**
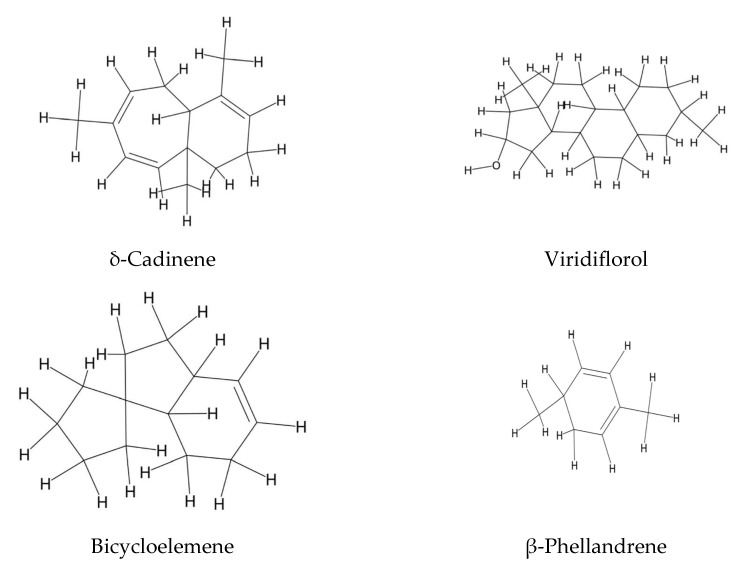

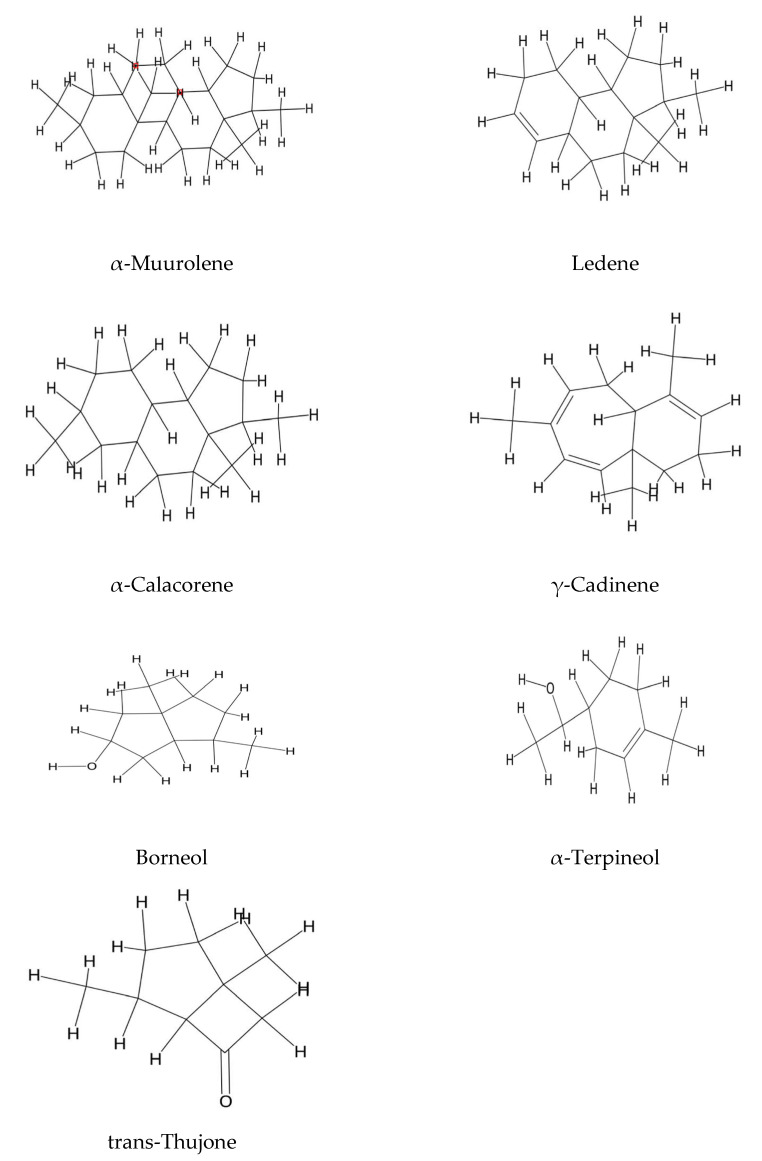
Sesquiterpenes and terpenes from the essential oil of *Echium* spp.

Figure 6 summarizes the major sesquiterpenes found in the essential oil of *E. amoenum*, which include cadinene (24.3%), viridiflorol (4.9%), β-muurolene (4.5%), ledene (3.8%), α-calacorene (3.0%), and γ-cadinene (2.9%) [85]. Similarly, the essential oil derived from the aerial parts of *E. humile* is characterized by the presence of bicycloelemene (15.9%), pentacosane (8.4%), p-cymen-8-ol (5.8%), β-phellandrene (4.9%), and trans-thujone (4.1%) [85].

These findings collectively highlight the diverse phytochemical landscape of *Echium* species. In particular, the presence of antioxidant steroids, bioactive monoterpenes and sesquiterpenes, and high levels of essential polyunsaturated fatty acids underscores the medicinal and nutritional relevance of this genus. The compositional variability across different species and plant parts suggests that *Echium* holds promise as a multipurpose genus for pharmaceutical applications [87,88,89].

### 2.4. Pyrrolizidine Alkaloids in Echium Species: Toxicity, Challenges and Mitigation-Strategies

Pyrrolizidine alkaloids (PAs), a class of toxic secondary metabolites, have been consistently detected across various genera within the Boraginaceae family, including *Echium*, *Symphytum*, and *Heliotropium*. Analytical techniques such as HPLC-ESI-MS and NMR spectroscopy have confirmed the presence of multiple hepatotoxic and genotoxic PA compounds such as echimidine, echimiplatine-N-oxide, and their isomers in methanolic extracts of several *Echium* species. In contrast, non-polar extracts, such as those obtained using hexane, showed no detectable PA content, indicating solvent-dependent variation in toxic compound recovery. These findings have raised considerable concerns about the safety of PA-containing medicinal plants and their derivatives in food and pharmaceutical products [90].

Approximately 600 plant species identified as containing toxic PAs belong to the family Boraginaceae. This family, which includes numerous melliferous (honey-producing) plants, encompasses all species within the genera *Echium* and *Symphytum* of the subfamily Boraginoideae, as well as the genus *Heliotropium* of the subfamily Heliotropioideae, and contains toxic PAs such as echimidine, lycopsamine, and vulgarine [91]. Toxic PAs are capable of entering the food chain and have been identified in a wide range of food and plant-based products such as cereals, tea, honey, herbal remedies, spices, and dietary supplements, posing potential health risks to consumers [92,93]. Chronic or excessive exposure to PAs has been associated with severe pathological outcomes, such as pulmonary hypertension, myocardial hypertrophy, renal dysfunction, and in extreme cases, mortality [94,95].

Due to the presence of toxic PAs across all species within the Boraginaceae family, there are increasing concerns regarding the safety of herbal medicinal products derived from these plants. In response to this issue, the General Statement on the Use of Herbal Medicinal Products Containing Toxic and Unsaturated PAs has established a Tolerable Daily Intake (TDI) of 0.35 µg/day for adults [96]. This threshold represents the maximum permissible daily exposure, based on toxicological risk assessments, aimed at minimizing the potential health risks associated with chronic ingestion of these hepatotoxic and genotoxic compounds.

Furthermore, the German pharmaceutical industry has established a comprehensive and practical code of practice aimed at minimizing the presence of toxic alkaloid compounds. This protocol addresses contamination risks at all critical stages of the production chain, including cultivation, harvesting, inspection of raw materials, pharmaceutical processing, and final product release, ensuring enhanced safety and quality control throughout [97,98].

High-performance liquid chromatography coupled with electrospray ionization mass spectrometry (HPLC-ESI-MS) revealed the presence of PAs such as pyrrolizidine N-oxides, tertiary pyrrolizidine alkaloids, leptanthine-N-oxide, echimiplatine-N-oxide, leptanthine, and echimiplatine in the methanolic extract of *E. vulgare*, whereas these toxic compounds were not detected in the hexane extract [43]. Notably, analysis of the methanolic extract derived from the leaves and flowers of *E. plantagineum* with HPLC-ESI-MS led to the identification of three distinct pyrrolizidine N-oxide alkaloids, such as 3′-*O*-acetylechiumine-*N*-oxide, echimiplatine-*N*-oxide, and echiuplatine-*N*-oxide [99]. HPLC-ESI-MS, which is considered one of the fastest and most reliable analytical techniques for profiling pyrrolizidine alkaloids, was particularly effective in detecting these compounds in *E. plantagineum*. Furthermore, using nuclear magnetic resonance (NMR) spectroscopy, the major PAs were identified as echimidine and a tigloyl isomer of echimidine in two other species, *E. setosum* and *E. vulgare* [100].

Given the widespread presence and toxicological implications of PAs in the Boraginaceae species, there is a pressing need for stricter quality control, improved regulatory frameworks, and continued development of sensitive detection methods.

## 3. General Applications, Medicinal Uses, and Pharmacological Studies of *Echium* spp.

Ethnobotanical surveys conducted in various regions (Table 1) confirm the traditional use of *Echium* species, particularly *E. italicum* and *E. amoenum*, for treating infections, inflammation, ulcers, skin conditions, and respiratory ailments. Additionally, in vivo experiments have demonstrated that chloroform root extracts of *E. italicum*, rich in naphthoquinones, significantly promote collagen synthesis and wound healing. Furthermore, essential oils derived from species within the Boraginaceae family are gaining recognition for their industrial relevance, especially in the pharmaceutical, cosmetic, and food sectors. Additionally, in vivo experiments have demonstrated that chloroform root extracts of *E. italicum*, rich in naphthoquinones, significantly promote collagen synthesis and wound healing. Furthermore, essential oils derived from species within the Boraginaceae family are gaining recognition for their industrial relevance, especially in the pharmaceutical, cosmetic, and food sectors.

In *E. italicum*, one of the major phenolic secondary metabolites released from its hairy roots in response to biotic and abiotic stress is shikonins, which are recognized for their antihistamine properties [101]. In fact, the shikonins extracted from the epidermal tissues of *E. italicum* have been identified in recent pharmacological studies not only for their antihistaminic properties, particularly beneficial for individuals with allergies, but also for their notable anticancer and anti-HIV activities [102,103,104]. Moreover, an additional recognized therapeutic effect of this compound includes the treatment of burns and eczema, neuroprotection, as well as antimicrobial, antithrombotic, and anti-inflammatory activities [102,103,104,105,106,107,108,109].

Furthermore, ethnobotanical studies conducted in the Antalya region of Turkey have identified three additional species from the Boraginaceae family, such as *E. italicum*, *E. vulgare* L., and *E. russicum* J.F. Gmellin, for their anti-ulcer, anti-inflammatory, and vulnerary activities [18].

Additionally, a traditional ethnobotanical study conducted in Bilecik Province, Turkey, identified *E. plantagineum* as a widely used medicinal plant for treating infections and skin disorders [19]. A recent study conducted on four *Echium* species, *E. italicum* L., *E. vulgare* L., *E. angustifolium* Miller., and *E. parviflorum* Moench, across different provinces of Turkey revealed that *E. italicum* contains higher levels of shikonin and its derivatives compared to the other species. These compounds represent the active ingredients in several pharmaceutical formulations [110].

Additionally, in vivo studies conducted in Turkey on *E. italicum* reported that the chloroform root extract of this species, due to its content of naphthoquinones, significantly accelerated wound healing in mice. Specifically, the treatment led to an increased level of hydroxyproline protein in the granulation tissue, indicating enhanced collagen turnover during the healing process [10]. An ethnobotanical survey conducted in Italy reported that *E. italicum* is traditionally used for the treatment of five different health conditions, making it one of the commonly used medicinal plants in the region [111] (Table 1). Similarly, an ethnobotanical study conducted in Iran, Alamut, reported that *E. amoenum*, Fisch. And May. Ahvazi 637 (IMPH), is the only species from the Boraginaceae family recognized for its traditional medicinal use [6]. The aqueous extract of *E. amoenum*, collected from Ardabil Province in Iran, has demonstrated antimicrobial activity against *S. aureus* (ATCC 8327) [37]. This finding supports the traditional use of the plant in treating fever and the common cold. Notably, *E. amoenum* has shown efficacy in treating neuropsychiatric disorders, such as anxiety and depression [30,87]. Multiple preclinical and clinical studies have highlighted the diverse therapeutic potential of its extracts, particularly the aqueous and methanolic forms. Clinical trials have demonstrated that oral administration of aqueous extracts of *E. amoenum* for 6 to 8 weeks significantly improves symptoms in patients with depression, anxiety, and obsessive–compulsive disorder. In vivo studies have further supported its neuroprotective potential, showing anticonvulsant effects when methanolic extracts are administered before picrotoxin-induced seizures and notable analgesic activity when given prior to nociceptive testing, with clinical trials supporting these claims [32,34,112,113]. They found that *E. amoenum* syrup was more effective than citalopram with fewer complications [112]. This highlights its potential as a natural, well-tolerated alternative for mood regulation.

In addition to its neuropsychiatric applications, a recent clinical trial revealed that a single oral formulation combining *E. amoenum* extract with chamomile and vitamin B6 significantly reduced the severity of premenstrual syndrome (PMS) symptoms, further supporting its use in women’s health [87].

Essential oils of aromatic plants, extracted from various parts such as leaves, stems, bark, seeds, fruits, roots, and plant exudates, contain a mixture of chemical compounds that are widely used in industries such as perfumery, food production, and pharmaceuticals [114]. Among plant families, Asteraceae, Lamiaceae, and Apiaceae are the most prominent sources of these essential oils [115]. Within the Boraginaceae family, certain species are considered industrially significant due to their production of essential oils. Notably, three species of borage identified in the Cape Verde region have been recognized as important sources of essential oils [115].

The diverse therapeutic potential and traditional applications of *Echium* species, particularly *E. italicum* and *E. amoenum*, highlight their value as candidates for further pharmacological research and pharmaceutical development. Future studies should focus on standardizing extraction methods, characterizing bioactive compounds such as shikonins and essential oils, and validating traditional claims through clinical trials to enable the safe and effective integration of these plants into modern therapeutic protocols.

**Table 1:** Medicinal uses of various *Echium* spp. and parts of plant.

Species	Medicinal Uses	Part of Plant	Extraction Techniques	Country
*Echium italicum* L.	Allergic disease	Hairy roots	ND^1^	ND^1^ [101]
*Echium italicum* L.	Wounds, anti-inflammatory	Leaf, root	Poultice	Turkey [18]
*Echium vulgare* L. and *E. russicum* J.F. Gmellin	Vulnerary	Root	Poultice	Turkey [18]
*Echium vulgare* L.	Wound healing, ulcer, bruising, pulled muscles, ligaments and sprains	Root	Ethanol and chloroform	Turkey [110]
*Echium vulgare* L.	Blood purification, heal wounds, tonic, diaphoretic, diuretic, astringent, treatment of snakebite	Leaves and flowering stems	Methanol and ethanol	East Serbia [80]
*Echium italicum* L. (PORUN—ADN 3851) BORAGINACEAE.	Depurative, diaphoretic, diuretic, emollient for healing respiratory infections	Aerial part	Decoction	Italy [111]
*Echium amoenum*, Fisch. And May. Ahvazi 637 (IMPH)	Demulcent, anti-inflammatory and analgesic, especially for common cold, pneumonia, anxiolytic, sedative, and other psychiatric symptoms, including obsession	Flower	Brewed	Iran [6]
*Echium amoenum* (F.M.)	Infectious diseases and for controlling fever	Flower	Aqueous extraction	Iran [37]
*Echium vulgare* L.	Antitussive, aphrodisiac, demulcent, diaphoretic, diuretic, pectoral, vulnerary, efficacious in the treatment of snake bites, for cleaning up the blood and healing wounds.	Leaves and flowering stems	ND^1^	Serbia [21]
*Echium Italicum* L.	Diuretic, sweet, and sedative	aerial parts	Decoction	Turkey [14]
*Echium italicum* L., *Echium vulgare* L. and *Echium angustifolium* Miller	Wound healing	Root and Aerial parts	Hydroalcoholic extract	Turkey [10]
*Echium plantagineum*	Diuretic and diaphoretic,	Aerial part	Decoction	Turkey [19]
*Echium Arabicum*. R. Mill	Antiplasmodial and antitrypanosomal activity	Aerial parts	Methanolic extract	Saudi Arabia [116]
*Echium flavum* Desf	Antiseptics and wound healing for ulcers and herpe	Root	Mashed or fried in olive oil	Spain [117]
*Echium vulgare* L.	Diuretic	Leaves	Aqueous EtOH, through maceration	Italy [118]
*Echium amoenum* Fisch. & C.A.Mey.	Cold and flu	Flower	ND^1^	Iran [119]
*Echium amoenum*	Reduce anxiety	Flower	Aqueous EtOH	Iran [120]
*Echium amoenum* Fisch. et May.	Improve depression	Flower	Aqueous extract	Iran [30]
*Echium amoenum*	Alleviate PMS symptoms		Aqueous extract	Iran [87]
*Echium italicum* L.	Sedative, Burns, Rheumatism, Uterus infection	Flower, root	Infusion, Cataplasm, Decoction	Iran [121]
*Echium amoenum* Fisch. & C.A.	Sore throat, nerve system relaxant, digestive	Therophytes, Aerial parts	Infusion, Decoction	Iran [3]
*Echium amoenum Fisch* and *May. Ahvazi 637* (*IMPH*)	Sedative, exhilarating, diuretic, analgesic, antioxidant, anxiolytic, diaphoretic	Flowers	Infusion	Iran [5,6,12,13,122]
*Echium hipernopicum*	Dietetic	Seed oil	Essential oil	Cape Verde [115]
*Echium stenosiphon*	Cough syrup	ND	Essential oil	Cape Verde [115]
*Echium vulcanorum*	Dietetic	Seed oil	Essential oil	Cape Verde [115]
*Echium* vulgare L.	As a revitalizing, anti-inflammatory, cough-relieving, asthma-relieving and phlegm-resolving agent, dry colds, biliary diseases, depression, heart and brain defciency syndromes, palpitations, insomnia	Flower	ND^1^	China [123]

ND^1^ = Not Defined.

## 4. Bioactivities of the *Echium* Genus

### 4.1. Antimicrobial Activity

Various *Echium* species, particularly *E. amoenum* and *E. italicum*, have garnered increasing scientific interest due to their rich content of bioactive compounds. In recent years, several in vitro studies have explored their antiviral, antibacterial, and antifungal activities, revealing promising potential for the development of novel plant-based therapeutics. The aqueous extract of *E. amoenum L*. flowers exhibited potent antiviral properties by inhibiting viral replication and proliferation at concentrations below 400 μg/mL [36]. Furthermore, both aqueous and ethanolic extracts demonstrated substantial antimicrobial activity against bacterial strains commonly associated with foodborne illnesses and spoilage [124]. The seed oil of *Echium amoenum* Fisch. & C.A.Mey revealed notable antimicrobial efficacy against *Candida albicans* and *Pseudomonas aeruginosa*, underscoring its potential as a natural therapeutic agent for managing cutaneous and mucosal infections caused by opportunistic pathogens [125]. Conversely, the aqueous extract at low concentrations did not display antibacterial effects, and its activity remained unaltered under varying pH conditions [37]. Remarkably, the methanolic flower extract showed effective antibacterial activity against Acinetobacter baumannii, positioning it as a potential alternative to colistin [126]. Among various extracts tested, only the ethyl acetate extract demonstrated significant inhibitory activity, particularly against Gram-positive bacteria. The ethyl acetate extract of *Echium arenarium* (*Guss*) exerted strong antibacterial effects against Bacillus cereus (ATCC 14579) and Staphylococcus aureus (ATCC 2592), while aqueous and ethanol extracts were largely inactive. [45].

Phytochemical analyses identified luteolin-7-O-glucoside and myricetin as key phenolic constituents contributing to the observed antibacterial activity, particularly against *S. aureus*, with a minimum inhibitory concentration (MIC) of 62.5 μg/mL. In comparison, quercetin exhibited weaker effects, with MIC values ranging from 125 to 250 μg/mL [45].

In the case of *E. serbicum* L., antibacterial assays demonstrated that all plant parts, including the root, stem, flower, and leaves, exhibited notable antibacterial activity against Gram-positive bacteria. While the leaf and stem extracts did not show sensitivity against Gram-negative strains, the root and flower extracts displayed partial sensitivity toward Gram-negative bacteria and overall exhibited stronger effects against Gram-positive strains. These findings are consistent with previous studies suggesting that phenolic compounds and other plant secondary metabolites generally exert greater antibacterial effects on Gram-positive bacteria compared to Gram-negative ones [127].

Studies on *E. italicum* L. oil confirmed a dose-dependent enhancement in antibacterial activity, with increasing inhibition zones at concentrations of 250, 500, 1000, 2000, 4000, and 8000 μg/disk [84]. Similarly, recent evaluations of its extracts showed increased antimicrobial effects at higher concentrations (25, 50, 75, and 100 mg/mL) [128]. Among these, the methanol extract at 100 mg/mL produced the strongest inhibition, forming zones of 14 ± 2 mm against *S. aureus* and 14 ± 1.5 mm against *E. coli*. In contrast, the hexane extract demonstrated peak antifungal activity against *Candida* at 50–100 mg/mL, with an inhibition zone of 15 ± 0.2 mm. Additionally, aqueous extracts of *E. amoenum* Fisch. & C.A.Mey at a dose of 5 mg exhibited 50% growth inhibition against *S. aureus* [35], as summarized in Table 2 (as shown in Table 2).

Collectively, the findings from various in vitro investigations underscore the potent antimicrobial potential of *Echium* species, particularly through methanolic, ethanolic, and seed oil extracts. Notably, ethyl acetate extracts demonstrated strong inhibitory effects against Gram-positive bacteria, while key phenolic compounds such as luteolin-7-O-glucoside and myricetin were identified as primary contributors to the observed bioactivity. These results suggest that *Echium* holds substantial promise as a natural source for developing alternative antimicrobial agents, especially in the face of rising antimicrobial resistance and the urgent need for novel therapeutic strategies [127].

**Table 2:** Antimicrobial activity of *Echium* spp.

Species	Virus/Bacteria	Part of Plant	Extract	Assay	Country
*Echium amenum* L.	herpes simplex virus type I (HSV-1, KOS strain) and Hep-2	Flower	Aqueous	Cytopathic effect inhibition assay (CPE)	Iran [36]
*Echium amenum* L.	*Staphylococcus aureus* (ATCC 25913), *Listeria monocytogenes* (ATCC 19117), *Escherichia coli* (ATCC 8739), *Yersinia enterocolitica* (ATCC 9610), and *Salmonella typhimurium strain* (ATCC 14028)	Flower	Aqueous and ethanolic	Disk diffusion method, and MIC^1^	Iran [124]
*Echium amoenum* Fisch. & C.A.Mey.	methicillin-resistant *Staphylococcus aureus* (MRSA), *Staphylococcus epidermidis*, *Pseudomonas aeruginosa*, *Candida albicans*, and *Aspergillus niger*	Seed	Chloroform and methanol	MIC	Iran [125]
*Echium amoenum* Fisch. & C.A.Mey.	*Staphylococcus aureus* 8327	Flower	Aqueous	Agar-well diffusion method and MIC	Iran [37]
*Echium amoenum* Fisch. & C.A.Mey.	*Acinetobacter baumannii*	Flower	Methanolic	disk diffusion method	Iran [126]
*Echium arenarium* (*Guss*)	*Bacillus cereus* ATCC 14579, *Listeria monocytogenes* ATCC 19115, *Staphylococcus aureus* ATCC 25923, methicillin-resistant *Staphylococcus aureus* (MRSA), *Enterrococcus faecalis* ATCC 29212, *Escherichia coli* ATCC 35214, *Pseudomonas aeruginosa* ATCC 27853, and *Klebsiella pneumonia* CIP 104727	Aerial part	Water, cyclohexane, dichloromethane and ethyl acetate	Disk diffusion method and MIC	Tunisia [45]
*Echium italicum* L.	*Bacillus subtilis*, *Staphylococcus aureus*, *Escherichia coli*, *Salmonella typhi*, *Pseudomonas aeruginosa*, *Aspergilus niger* and *Candida albicans*	Flowering aerial parts	Hydrodistillation	Disk diffusion method and MIC	Iran [84]
*Echium italicum* L.	*Bacillus megaterium*, *Escherichia. coli*, *Staphylococcus aureus*, *Candida albicans*, and *Klebsiella pneumonia*	Flower, stems, and leaves	Methanol and hexane	Agar-well diffusion method and MIC	Turkey [128]
*Echium serbicum* L.	*Bacillus mycoides* ATCC 6462, *Bacillus subtilis* ATCC 6633, *Enterococcus faecalis* ATCC 29212, *Micrococcus luteus* ATCC 1024, *Micrococcus lysodeikticus* ATCC 4698, *Staphylococcus aureus* ATCC 25923, *Staphylococcus epidermidis* ATCC 14990, *Escherichia coli* ATCC 25922, *Klebsiella pneumoniae* ATCC 13883, *Pseudomonas aeruginosa* ATCC 19429	Flowers, leaves, stems, and roots	Methanol	MIC	[127]
*Echium italicum* L.	*Bacillus subtilis*, *Staphylococcus aureus*, *Escherichia coli*, *Salmonella typhi*, *Pseudomonas aeruginosa*, *Aspergilus niger* and *Candida albicans*	Aerial part	Essential oil	Disk diffusion method	Iran [84]
*Echium amoenum* Fisch. & C.A.Mey.	*Staphylococcus aureus*	Flower	Aqueous	Agar-well diffusion method	Iran [35]

MIC^1^ = Minimal Inhibitory Concentration.

### 4.2. Antioxidant Activity

Antioxidant compounds derived from medicinal plants play a critical role in mitigating oxidative stress induced by free radicals and have attracted considerable attention in pharmaceutical and nutritional research. Among these, various species of the *Echium*, particularly *E. amoenum*, have been extensively studied due to their high content of phenolic compounds. This section summarizes the findings of previous studies on the antioxidant properties of *Echium* species, with a focus on the influence of solvent type, extraction techniques, drying conditions, and ecological factors on the retention and enhancement of antioxidant activity.

In *E. amoenum*, anthocyanins such as cyanidin-3-glucoside [1] have shown a significant protective effect on human endothelial cells exposed to oxidative stress, especially at higher concentrations (100–1000 µMs) [23].

Aqueous extracts displayed stronger antioxidant activity than acetone extracts, emphasizing the influence of solvent type and polarity on compound efficacy [129]. Antioxidant activity varies based on the type and concentration of the solvent used [130].

Noteworthy, recent reports indicated that in the case of *E. amoenum*, a species commonly reported in Iran, drying conditions, particularly temperature, are critical for maintaining total phenolic content (TPC), total flavonoid content (TFC), anthocyanins, and overall antioxidant capacity. A drying temperature of 60 °C and an air velocity of 0.86 m/s are optimal for preserving phenolic compounds [131].

In *E. vulgare*, the efficiency of phytochemical extraction, particularly polysaccharides with demonstrated antioxidant and anti-Listeria activity, has been shown to depend on several parameters, including extraction time (73.8 min), microwave power (769.2 W), extraction temperature (42.3 °C), and the water-to-raw-material ratio (61.4 mL/g) [24].

Notably, drying conditions such as 60 °C and 0.86 m/s air velocity are optimal for preserving phenolic compounds.

In a study investigating the influence of geographical location on the antioxidant activity of *E. italicum*, plant samples were collected from three provinces in Turkey: Gaziantep, Hakkari, and Mersin. The results indicate that samples collected from the Mersin region exhibit significantly higher antioxidant activity, as well as elevated levels of Total Oxidant Status (TOS) and the Oxidative Stress Index (OSI), in comparison to samples obtained from the other regions. These findings highlight the significant impact of environmental and geographical factors on the antioxidant capacity of *E. italicum* [132]. Ethanol and acetone proved to be the most effective solvents for extracting antioxidant compounds [133]. Furthermore, ultrasound-assisted extraction (UAE) conducted under optimized conditions, specifically at 41.84 °C for 45 min, with a liquid-to-solid ratio of 10:0.78, resulted in the highest recovery efficiency for shikonins and phenolic antioxidants [134].

Numerous studies have established a direct correlation between antioxidant activity and the presence of phenolic compounds [135]. Nevertheless, some research indicates that such a relationship may not exist [136]. This disparity may arise because not all plant phenolic compounds exhibit antioxidant properties. The antioxidant activity is influenced by the position of hydroxyl groups on aromatic rings and the oxidation state of unpaired electrons within the phenolic structure [137]. This disparity may arise because not all plant phenolic compounds exhibit antioxidant properties. The antioxidant activity is influenced by the position of hydroxyl groups on aromatic rings and the oxidation state of unpaired electrons within the phenolic structure [137]. For example, among *E. arenarium* (*Guss*) extracts, ethyl acetate extracts exhibited the strongest antioxidant activity despite lower overall phenolic content, suggesting compound-specific effects [45] (Table 3).

The antioxidant activity of *E. amoenum* flower decoction is attributed to phenolic acids like rosmarinic acid and various flavonoids [12]. Rosmarinic acid, a significant compound in the Boraginaceae family, exhibits stronger antioxidant activity than vitamin E. It plays a crucial role in scavenging free radicals and adicals [138], inhibiting low-density lipoprotein oxidation [138], suppressing oil oxidation [139], suppressing arachidonate metabolism [140], preventing hemolysis [141], and exhibiting hyaluronidase and h-hexosaminidase activities [142]. Due to the presence of phenolic compounds, glycerol (CHGLY)-based natural deep eutectic solvent (NADES) demonstrates superior antioxidant activity compared to methanol, ethanol, and water in extracts from *E. amoenum* flower species [53]. Among the three types of *E. amoenum* flower extracts, the ultrasonic extract, alkaline fraction, and polyphenol extract exhibited the highest antioxidant activities, respectively [143].

The phenolic compounds in plants can stabilize metal ions that catalyze hydrogen peroxide decomposition, thereby preventing oxidative reactions [144,145]. In *Echium* spp. flower extracts, phenolic compounds such as apigenin, luteolin-7-O-rutinoside, apigenin-7-O-rhamnoside, and quercetin-3-O-rhamnoside in ethyl acetate and butanol extracts have been identified as key contributors to strong antioxidant activity [11].

**Figure 7:**
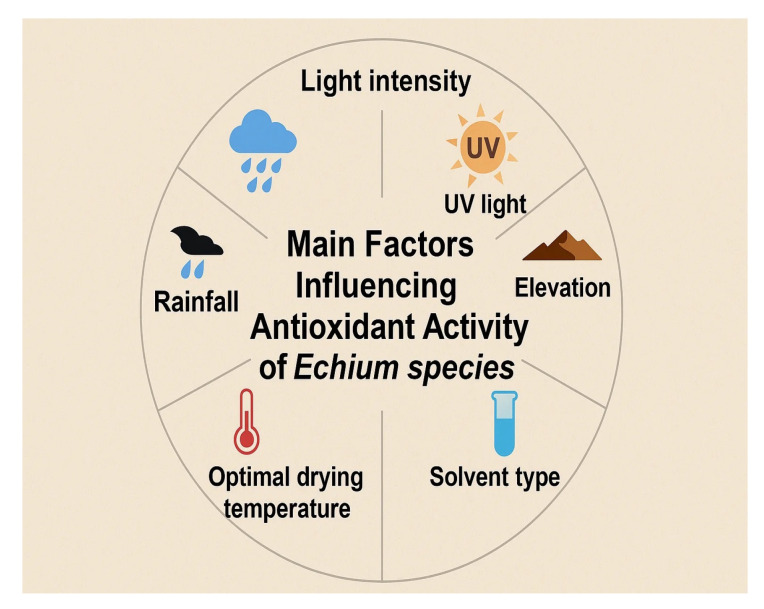
Main factors enhancing the antioxidant activity of *Echium* spp.

Important ecological factors like temperature, rainfall, UV light, light intensity, altitude, longitude, and latitude significantly influence the concentration of phenolic compounds (Figure 7).

Plants growing at higher altitudes have been reported to contain more phenolic and flavonoid compounds than those at lower altitudes [146,147,148]. For instance, extracts from *Echium* spp. seeds and stems in the Bushehr and Ramsar regions exhibited higher phenolic content and stronger antioxidant activity compared to borage seeds from Alamut and Boumehen [149].

Drying conditions also affect the preservation of bioactive compounds in *E. amoenum* flowers. High temperatures combined with moderate airspeed were found to retain bioactive compounds and enhance antioxidant activity [131]. Similarly, studies on lemon myrtle leaves indicate that freeze-drying preserves the highest phytochemical and antioxidant properties among six drying methods. However, due to its high energy consumption, microwave drying is preferred in the industry for its energy efficiency while preserving antioxidant activity and phytochemical content [150].

The optimal methods for preserving phytochemical compounds in borage plants are decoction, infusion, and hydromethanolic extraction [59]. The species of *E. rubrum* and *E. vulgare* exhibited the highest hydroxyl radical scavenging activity and iron chelating ability. Among them, *E. vulgare* was reported to have a higher flavonoid content [21]. When comparing the antioxidant activity of methanol and hexane extracts of *E. italicum*, the hexane extract demonstrated an IC_50_ value of 63.3 μg/mL, while the methanol extract exhibited a significantly lower IC_50_ value of 20.7 μg/mL (66). This indicates that the methanol extract contains a higher concentration of compounds capable of neutralizing free radicals, which accounts for its superior antioxidant activity compared to the hexane extract [128].

Among the extracts analyzed from *E. humile* Desf., the ethyl acetate and methanol extracts exhibited the highest polyphenol content, as determined by HPLC-MS analysis. Among the identified phenolic compounds, p-coumaric acid was the most abundant, followed by cirsiliol [151].

Among the extracts of *E. vulgare* (chloroform, ethyl acetate, ethanol, acetone, and petroleum), the ethanolic extract exhibited the highest antioxidant activity with an IC_50_ value of 49.48 ± 1.33 µg/mL, followed by the acetone extract (50.50 ± 1.10 µg/mL) and the chloroform extract (51.34 ± 1.06 µg/mL). In the case of *E. italicum*, the acetone extract demonstrated the most pronounced inhibition of lipid peroxidation, with an IC_50_ value of 42.54 ± 1.13 µg/mL, followed by the chloroform extract (43.29 ± 1.20 µg/mL) and the ethanol extract (44.56 ± 1.29 µg/mL) [152] (Table 3).

**Figure 8:**
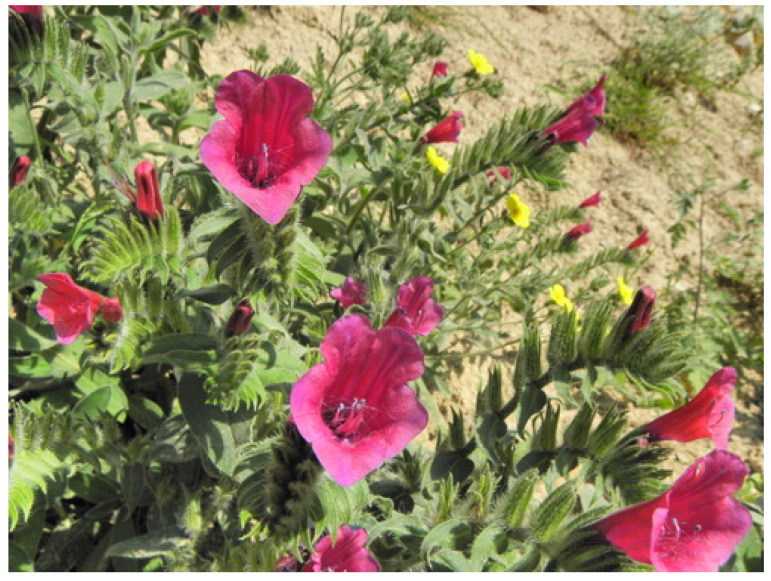
*E. amoenum* species (https://www.inaturalist.org/photos/288847187, accessed on 14 July 2025).

*E. amoenum*, shown in Figure 8, is the most studied species regarding its antioxidant properties.

Notably, the roots of *Echium* species have consistently exhibited the highest antioxidant activity, which can largely be attributed to their elevated levels of phenolic compounds [153].

**Table 3:** Antioxidant activity of *Echium* spp.

Scientific Name	Part of Plant	Extract	Type of Study	Reference
*Echium* Fisch. & C.A. Mey.	Petals	Dichloromethane	Ferric Reducing Antioxidant Power Assay (FRAP)	[23]
*Echium amoenum*	Petals	Ethanol, methanol, acetone, ethanol 80% and water extracts	1,1 Diphenyl 2-Picryl Hydrazyl (DPPH)	[129]
*Echium arenarium* (*Guss*)	Aerial part	Ethyl acetate extracts cyclohexane, dichloromethane, and water	1DPPH; β-carotene bleaching assay	[45]
*Echium amaenum* Fisch & C.A. Mey	Flowers	Decoction	blood total antioxidant capacity (TAC), lipid peroxidation (LPO) and total thiol (SH) molecules	[12]
*Echium amoenum*	Flower	Ethanol, water, methanol and a choline chloride and glycerol (CHGLY)	DPPH and FRAP	[53]
*Echium amoenum* Fisch & C.A. Mey	Petals	Ultrasonic extract, Polyphenol fraction, and Alkaloid fraction	DPPH	[143]
*Echium pycnanthum* Pomel	Roots	Hydromethanolic	DPPH, b-carotene bleaching test, 1,1-diphenyl-2-picrylhydrazyl, 2,2-azino-bis-3-ethyl benzthiazoline-6-sulfonic acid (ABTS), Chelating effect on ferrous ions, Iron reducing power	[145]
*Echium sericeum* (Vahl)	Aerial parts	Chloroform, ethyl acetate, and n-butanol, the ethanolic extract	DPPH	[11]
*Echium amoenum* Fisch & C.A, *Echium italicum* L.	Leaf, stem, and seed	Aqueous EtOH	DPPH and FRAP	[149]
*Echium amoenum* Fisch & C.A	Petal	Methanol and 70% acetone	DPPH	[131]
*Echium italicum* L.	Root	*n*-Hexane	DPPH	[134]
*Echium amoenum*	Flower	Decoction, infusion, methanolic, and hydroalcoholic extract	DPPH, FRAP, ABST	[59]
*Echium vulgare* L. and, *Echium rubrum* L.	Aerial parts	Methanol and ethanol	total antioxidant capacity, DPPH free-radical scavenging, the inhibitory activity toward lipid peroxidation, Fe^3+^- reducing power, Fe^2+^- chelating ability, and hydroxyl radical scavenging activity	[21]
*Echium italicum* L.	Flowers, stems, and leaves	Methanol and hexane	DPPH	[128]
*Echium italicum* L	Aerial parts	Chloroform, ethyl acetate, ethanol, acetone, and petroleum ether extracts	DPPH, total phenolic content, flavonoid content, inhibitory activity against lipid peroxidation, and hydroxyl radical scavenging activity	[133]
*Echium serbicum* L.	Flowers, leaves, stems, and roots	Methanol	DPPH, ABTS, reducing power activity, Inhibition of lipid peroxidation	[127]
*Echium humile* Desf	Aerial part	Hexane, dichloromethane, ethyl acetate, methanol, and aqueous	DPPH, ABTS, FRAP, and TAC	[151]
*Echium vulgare* L. and *Echium italicum* L.	Flower	Chloroform, ethyl acetate, ethanol, acetone, petroleum	Determination of inhibition of lipid peroxidation by ammonium thiocyanate	[152]

### 4.3. Cytotoxicity Activity

A key determinant of the pharmacological efficacy of plant extracts is the polarity of the solvent used during extraction, which influences the spectrum of isolated compounds.

This study evaluates the cytotoxic potential of different solvent extracts, including methanol, hexane, chloroform, acetone, and ethyl acetate obtained from *Echium* spp., tested against various cancer cell lines and normal cell lines.

The methanolic extract of *E. italicum* demonstrated marked cytotoxic activity against the human breast cancer cell line MCF-7, with an IC_50_ value of 202.2 µg/mL. In contrast, the hexane extract exhibited substantially lower potency, yielding an IC_50_ of 853 µg/mL. These results suggest that the methanolic extract contains a higher concentration of bioactive anticancer constituents compared to the hexane fraction [128].

Among the tested extracts of *E. vulgare* L. and *E. italicum* L., those prepared using chloroform and acetone showed the most pronounced antiproliferative effects, particularly against the murine tumor fibroblast cell line (L2OB) [152].

At concentrations ranging from 1 to 100 µg/mL, the hexane extract derived from *E. italicum* seeds exhibited no significant cytotoxicity against either HepG2 (human hepatoma) or MCF-7 (human breast cancer) cell lines. However, at a concentration of 200 µg/mL, the hexane extract displayed a markedly enhanced cytotoxic effect against HepG2 cells, surpassing the activity of the methanolic extract. Interestingly, no inhibitory effect was observed on MCF-7 cells at any tested concentration of the hexane extract [154]. This implies that, in the case of HepG2 cells, the hexane fraction harbors compounds with greater cytotoxic potency than those present in the methanolic extract.

Similarly, hexane, dichloromethane, and ethyl acetate extracts from the flowering aerial parts of *E. amoenum* showed no cytotoxic effects on J774.1A murine macrophages at concentrations between 1 and 100 µg/mL. Nevertheless, a concentration-dependent response was observed, as the highest cytotoxicity was recorded at 200 µg/mL in J774.1A cells [155] (Table 4).

Therefore, evaluating the hexane extract in *Echium* species will be of significant importance in future studies, as it is likely to contain potent anticancer compounds.

In contrast, in *E. creticum*, among the aqueous, ethyl acetate, and methanolic extracts tested, only the methanolic extract demonstrated significant cytotoxic effects against breast cancer cells (MCF-7) [156]. Interestingly, in another study on the same species, *E. creticum*, both ethanolic and aqueous leaf extracts exhibited significant cytotoxicity against HeLa cancer cells. This activity was attributed to the presence of various phytochemical constituents, including alkaloids, tannins, coumarins, saponins, flavonoids, and polyphenols [157].

It has also been reported that methanolic extracts derived from the roots, flowers, and leaves of *E. serbicum* exhibit selective cytotoxicity against various cancer cell lines, HCT-116, SW-480, MDAMB-231, and normal MRC-5 cancer cells. This selective activity is primarily attributed to the presence of major bioactive constituents such as rosmarinic acid and chlorogenic acid [127].

**Figure 9:**
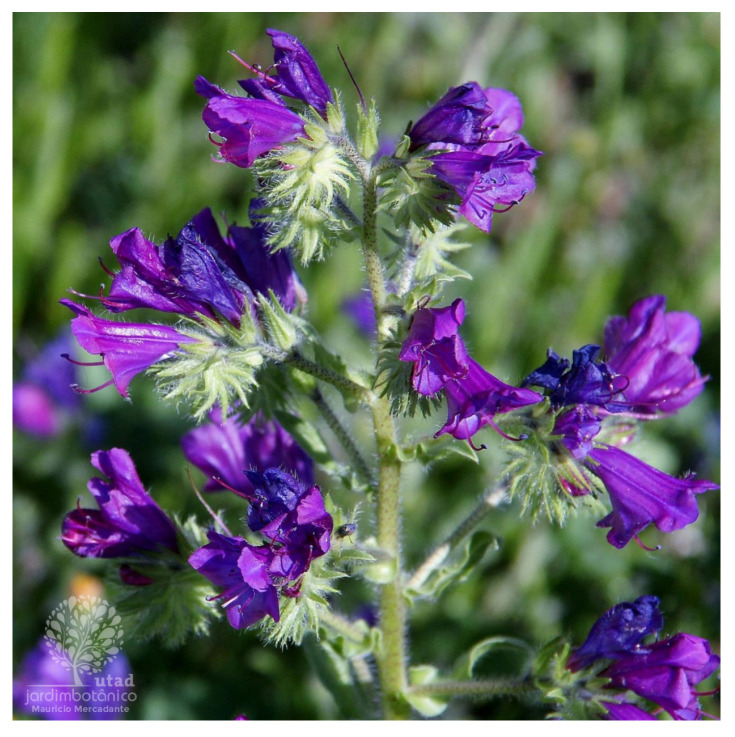
*E. plantagineum* in Portugal (https://jb.utad.pt/especie/Echium_plantagineum#imagem-14486, accessed on 14 July 2025).

A study conducted on honey extracts derived from *E. plantagineum* (Figure 9), collected in different regions of Portugal, revealed, through HPLC analysis, the absence of toxic pyrrolizidine alkaloids. Instead, the extracts were rich in phenolic compounds such as hydroxybenzoic acid, hydroxycinnamic acid, p-coumaric acid, caffeic acid, and quercetin. Cytotoxicity assays demonstrated that while the extracts had no adverse effects on normal MRC-5 fibroblast cells, they significantly reduced the viability of AGS gastric cancer cells [41].

These findings suggest that the phenolic compounds present in Portuguese *Echium* species exert selective anticancer effects, targeting tumor cells while sparing normal ones. This observation aligns with previous studies on other *E. serbicum* L. species, where phenolics were shown to decrease the viability of breast and colon cancer cells without harming normal cells [127].

The data presented underscore the critical role of solvent polarity in shaping the cytotoxic profile of *Echium* extracts. Collectively, these findings emphasize the necessity of strategic solvent selection to optimize the recovery of targeted anticancer compounds and to guide the development of effective plant-based therapeutics.

**Table 4:** Cytotoxicity activities of *Echium* spp.

Taxon	Extract	Biological Activity	Assay	Part of Plant	Ref.
*Echium italicum* L.	Methanol and hexane	HepG2, MCf-7	MTT	Seed	[154]
*Echium vulgare* L and *Echium italicum* L.	Chloroform, ethyl acetate, ethanol, acetone, and petroleum	Hep2, RD, L2OB	MTT	Flower	[152]
*Echium amoenum*	Hexane, dichloromethane, and ethyl acetate	J774.1A macrophage cell line	MTT	Flower	[155]
*Echium serbicum* L.	Methanol	HCT-116, SW-480, MDAMB-231 and MRC-5	MTT	Flowers, leaves, stems, and roots	[127]
*Echium plantagineum* L.	Methanol	AGS and MRC-5	MTT	Pollen	[41]
*Echium arenarium* Guss	Hydromethanolic	U266	MTT	Root and aerial parts	[45]
*Echium angustifolium* Mill	n-Hexane (nonpolar)	HCT116 and HEPG2	SRB	Aerial part	[158]
*Echium creticum*	Aqueous, ethyl acetate extracts, and methanol	MCF-7	MTT	Leaves and stem	[156]
*Echium italicum* L.	Methanol and hexane	HepG2 and MCF 7	MTT	Flowers, stems, and leaves	[128]

## 5. Conclusions

This review emphasizes the phytopharmaceutical significance of *Echium* species, which are characterized by a rich diversity of bioactive secondary metabolites, including flavonoids, naphthoquinone derivatives, terpenoids, and essential polyunsaturated fatty acids. Notable compounds such as luteolin-7-O-glucoside, kaempferol-3-O-neohesperidoside, and rosmarinic acid exhibit a broad spectrum of pharmacological effects, including antioxidant, anti-inflammatory, neuroprotective, and antimicrobial activities. The roots of several *Echium* species are particularly enriched with alkanins and shikonins, naphthoquinone compounds with well-documented wound-healing, collagen-stimulating, and anticancer properties. In addition, volatile constituents such as thymol, carvacrol, and pulegone contribute further to the antimicrobial and antifungal potential of these plants. The seed oil of *E. amoenum*, rich in alpha-linolenic acid (ALA), highlights the genus’s relevance in pharmaceutical, nutraceutical, and cosmeceutical applications.

The use of advanced analytical platforms, especially spectroscopic techniques, has significantly enhanced our ability to identify and characterize these metabolites across diverse plant matrices. However, the presence of PA toxic compounds associated with hepatotoxicity remains a critical safety concern. Therefore, future research should prioritize the development of optimized extraction protocols aimed at minimizing PA content while preserving the therapeutic efficacy of the extracts. Investigations into alternative plant parts and innovative processing strategies may also help balance efficacy with consumer safety.

Altogether, the phytochemical complexity of *Echium* species underscores their substantial biomedical potential and justifies further research to isolate, characterize, and understand the mechanisms of action of their active constituents. These efforts are essential for the rational development of safe and effective phytopharmaceuticals derived from this valuable genus. The observed antimicrobial, antioxidant, and cytotoxic activities of *Echium* extracts underscore their promise in the development of novel therapeutic agents. These findings support the need for further pharmacological and clinical research to fully explore and validate the medicinal potential of this versatile genus.

## Data Availability

The original contributions presented in this study are included in the article.
